# Epigenetic Regulation in Acute Myeloid Leukemia: Molecular Mechanisms and Clinical Implications

**DOI:** 10.3390/cancers18142203

**Published:** 2026-07-08

**Authors:** Jingru Xu, Georges Lacaud

**Affiliations:** Cancer Research UK, Stem Cell Biology Group, Cancer Research UK Manchester Institute, The University of Manchester, Wilmslow Road, Manchester M20 4BX, UK

**Keywords:** acute myeloid leukemia, epigenetics, DNA methylation, histone modification, chromatin remodeling, RNA-mediated epigenetics, targeted therapy, epigenetic therapy, immunotherapy

## Abstract

Acute myeloid leukemia (AML) is a heterogeneous hematologic malignancy characterized by a block of differentiation and uncontrolled expansion of myeloid progenitor cells. Standard treatment includes intensive induction chemotherapy, typically with cytarabine and anthracycline, followed by consolidation chemotherapy or allogeneic hematopoietic stem cell transplantation. However, these approaches are often associated with relapse and treatment-related toxicity. Accumulating evidence highlights a critical role for epigenetic dysregulation in driving disease initiation, progression, and therapeutic resistance. In this review, we examine an integrated framework of epigenetic regulation in AML, encompassing DNA methylation, histone post-translational modifications, chromatin remodeling, and RNA-mediated epigenetics. We discuss how alterations in key epigenetic regulators, such as DNMT3A, TET2, IDH1/2, EZH2, and histone-modifying enzymes, reshape the transcriptional and epigenetic landscape of leukemic cells. Particular emphasis is placed on epigenetically defined AML subtypes, including *NPM1*-mutated, *DNMT3A*-mutated, and *KMT2A*-rearranged AML, which illustrate distinct mechanisms of transcriptional and epigenetic dysregulation and confer unique therapeutic vulnerabilities. We further summarize current and emerging therapeutic strategies, ranging from conventional chemotherapy to molecularly targeted agents, epigenetic drugs, and immunotherapeutic approaches. Despite these advances, durable responses remain limited, highlighting the need to better understand epigenetic mechanisms to overcome resistance and improve patient outcomes.

## Simple Summary

Epigenetics is the study of heritable changes in gene expression that do not alter the underlying DNA sequence. In acute myeloid leukemia (AML), these regulatory mechanisms are frequently dysregulated, driving uncontrolled proliferation and blocking normal myeloid differentiation. This review systematically examines the principal layers of epigenetic regulation, including DNA methylation, histone modifications, chromatin remodeling, and RNA-mediated epigenetics. We describe how their disruption contributes to AML pathogenesis, with particular emphasis on how recurrent genetic lesions such as *DNMT3A* mutations and *KMT2A* (*MLL*) rearrangements shape the epigenetic landscape of the disease. We further discuss current therapeutic options for AML, with a focus on approved agents and ongoing clinical trials targeting epigenetic regulators.

## 1. Introduction

Hematopoiesis is a tightly regulated process in which hematopoietic stem cells progressively differentiate into all mature blood cells. Acute myeloid leukemia develops when this process is disrupted, leading to a block in myeloid differentiation and the abnormal expansion of immature progenitor cells. These leukemic blasts accumulate in the bone marrow, resulting in bone marrow failure and clinical symptoms such as anemia, infection, and bleeding. Clinically, AML is a life-threatening malignancy that predominantly affects older individuals, with a median age at diagnosis of around 68–70 years. Standard treatments include intensive chemotherapy and hematopoietic stem cell transplantation [1,2]. Despite improvements in treatment, the overall five-year survival rate remains low, and relapse occurs frequently, highlighting a pressing need to develop more effective therapies for AML. Genomic sequencing studies have advanced our understanding of AML biology and show that the mutational spectrum of AML spans multiple functional categories [3]. Notably, mutations affecting epigenetic regulators, including chromatin-associated factors and DNA methylation enzymes, represent a large proportion of AML mutations. This enrichment suggests that disruption of epigenetic regulation is a central feature of AML pathogenesis.

In broad terms, epigenetic regulation in AML operates across multiple, interconnected layers. At the DNA level, mutations in DNA methylation regulators such as *DNMT3A*, *TET2*, and *IDH1/2* disrupt normal methylation dynamics and collectively account for about 46% of AML cases [4]. Additional epigenetic layers include histone modifications, chromatin remodeling, and RNA-mediated mechanisms. A well-known example is *KMT2A* (also known as *MLL*), which frequently undergoes chromosomal rearrangements to form fusion proteins that drive histone methylation and activation of oncogenic transcriptional programs. Although key mutated and fusion-driven regulators in AML, such as KMT2A fusions, have been identified and functionally characterized, the roles of non-mutated epigenetic regulators remain largely unknown. Clarifying these non-mutated chromatin-associated factors may reveal shared dependencies and inform the development of broadly applicable therapeutic strategies. In this review, we examine the molecular mechanisms by which epigenetic regulation is disrupted in AML, with particular focus on how specific genomic alterations reshape the chromatin landscape and drive leukemic transformation. We further discuss the clinical implications of these epigenetic aberrations. This narrative review draws on PubMed searches for articles published up to May 2026. Search strategies combined “acute myeloid leukemia” with terms for the epigenetic regulators and modifications discussed here, including DNMT3A, DNMT1, TET2, IDH1/IDH2, KMT2A (MLL), DOT1L, menin (MEN1), p300/CBP, HDACs, BET proteins, the SWI/SNF complex, and the m6A machinery (METTL3, FTO, ALKBH5). Priority was given to mechanistic primary studies and recent clinical trials. Clinical trials were identified via ClinicalTrials.gov, and approved agents were verified against FDA records.

## 2. Hematopoiesis

Hematopoiesis is the biological process that generates all cellular components of the blood, encompassing the myeloid and lymphoid lineages. The myeloid lineage comprises erythroid cells, megakaryocytes, granulocytes, and monocytes, which are responsible for oxygen transport, hemostasis, and innate immunity. The lymphoid lineage gives rise to B cells and T cells, which mediate adaptive immunity, as well as natural killer (NK) cells, which contribute to innate immunity. These lineages originate from hematopoietic stem cells (HSCs), a functionally and molecularly heterogeneous population capable of self-renewal, multilineage differentiation and long-term quiescence. HSC fate decisions are governed by tightly regulated, multi-step differentiation processes, driven by intrinsic genetic and epigenetic programs as well as extrinsic signals from the microenvironment [5,6]. Traditionally, hematopoiesis has been described as a hierarchical process, in which HSCs give rise to multipotent progenitors (MPPs), which differentiate into lineage-restricted progenitors, including common myeloid progenitors (CMPs) for the myeloid lineages, and lymphoid-primed multipotent progenitors (LMPPs) for the lymphoid lineage [6,7]. These progenitor populations can be prospectively isolated by flow cytometry based on differential surface marker expression, and characterized functionally by colony-forming unit (CFU) assays and in vivo transplantation into irradiated mice to evaluate the self-renewal capacity and lineage potential [8]. However, single-cell RNA sequencing has revealed a continuous, rather than strictly hierarchical, trajectory of hematopoiesis [6,9,10]. Moreover, CRISPR-Cas9-based perturbation screens coupled with single-cell transcriptomics have demonstrated that disrupting key transcription factors leads to distinct lineage biases and altered differentiation trajectories. These findings support a revised model of ‘punctuated continuity,’ in which differentiation proceeds along a continuous developmental trajectory but is punctuated by discrete, regulator-driven transcriptional shifts that mark critical fate transitions [11,12].

## 3. Acute Myeloid Leukemia (AML)

The development of hematopoietic cells is orchestrated by tightly regulated genetic and epigenetic programs, and disruption of these mechanisms can lead to leukemogenesis. Leukemia is a hematological malignancy originating in the bone marrow, typically arising from HSCs or early progenitor cells, and is characterized by uncontrolled proliferation and impaired terminal differentiation [13]. Acute myeloid leukemia (AML) is the most common and most aggressive acute leukemia in adults [14]. It is distinguished by the clonal expansion of immature myeloid progenitor cells and typically arises from HSCs or early myeloid progenitors following genetic and epigenetic alterations. As a life-threatening hematologic malignancy, AML leads to bone marrow failure, commonly manifesting as severe anemia, thrombocytopenia with bleeding, and recurrent infections due to impaired production of functional erythroid, myeloid, and megakaryocytic cells [1,2]. AML diagnosis is based on the detection of ≥20% blasts in the bone marrow or peripheral blood, or the presence of specific AML-defining genetic abnormalities. This is followed by immunophenotypic classification using flow cytometry to characterize the blast population (e.g., CD13, CD33, CD117). Cytogenetic analysis and FISH are performed to identify chromosomal abnormalities, and molecular testing is carried out to detect gene mutations that further refine diagnosis and risk classification [2,15]. AML is predominantly a disease of the elderly, with a median age at diagnosis of 68–70 years. The overall five-year survival rate for AML across all age groups is about 30%, and the survival outcomes vary significantly by age, genetic abnormalities, disease stage and likelihood of relapse [1,2].

AML is driven by a diverse spectrum of genomic and epigenetic alterations that disrupt normal hematopoietic differentiation and promote uncontrolled proliferation. Integrative genomic profiling by The Cancer Genome Atlas (TCGA) Research Network has revealed that 99% of AML cases harbor at least one biologically significant somatic mutation, underscoring the importance of a comprehensive view of the genetic and epigenetic landscape in AML pathogenesis [16]. Specifically, the TCGA study performed integrative analyses, including whole-genome/exome sequencing, RNA and microRNA profiling, and DNA methylation, on 200 de novo AML samples. These driver mutations were classified into nine functional categories based on their biological roles: activated signaling pathways (59%, e.g., KRAS), DNA methylation regulators (46%, e.g., DNMT3A), chromatin modifiers (30.5%, e.g., KMT2A fusions), NPM1 mutations (27%), myeloid transcription factors (22%, e.g., CEBPA), transcription factor fusions (18%, e.g., RUNX1-RUNX1T1), tumor suppressors (16.5%, e.g., TP53), spliceosome genes (13.5%), and cohesin complex components (13%) [3,16]. These categories reflect the diverse molecular drivers that contribute to leukemogenesis and are increasingly used to guide therapeutic strategies in AML.

Different genomic classes of AML are associated with distinct risk profiles, resulting in variable clinical outcomes, and guiding therapeutic decision-making. The 5th edition of the World Health Organization (WHO) classification, published in 2022, refined AML subtypes by incorporating expanded molecular criteria, reflecting advances in both scientific understanding and clinical practice [17], paralleled by the International Consensus Classification (ICC) [18]. Accordingly, AML patients with different genomic alterations demonstrate diverse clinical behaviors and require tailored therapeutic strategies based on their underlying genetic features. Beyond genetic alterations, epigenetic dysregulation has emerged as a central hallmark of AML, contributing to disease initiation, progression, and therapeutic response. In AML, these processes are frequently disrupted, leading to aberrant transcriptional landscapes that promote leukemic cell self-renewal, block differentiation, and enhance survival.

A primary paradigm of this interface between genetic and epigenetic disruption is seen in NPM1-mutated AML. Nucleophosmin (NPM1) is a multifunctional protein that serves as a critical histone chaperone, facilitating chromatin assembly and genome stability [19]. *NPM1* mutations (*NPM1*^mut^) account for approximately one-third of AML cases, representing the most frequent genomic subgroup of the disease [15]. The mutant NPM1 protein lacks critical tryptophan residues at its C-terminus, which are essential for nucleolar retention, and acquires a novel nuclear export signal (NES) due to a frameshift mutation in exon 12. Together, these alterations lead to the aberrant cytoplasmic localization of the mutant NPM1 protein, in contrast to the predominantly nuclear localization of the WT form. The cytoplasmic mislocalization of mutant NPM1 disrupts chromatin stability and leads to aberrant transcriptional and epigenetic regulation, including the upregulation of leukemogenic gene expression programs [20,21]. A notable co-occurring mutation with *NPM1* involves *DNMT3A*, the gene encoding a DNA methyltransferase, which is present in approximately 50% of *NPM1*^mut^ AML cases [22]. Disruption of DNMT3A impairs de novo DNA methylation, leading to epigenetic dysregulation. In addition, the co-occurrence of *NPM1*, *FLT3*-ITD, and *DNMT3A* mutations also defines a frequent molecular subtype of AML, characterized by aggressive disease biology and resistance to conventional chemotherapy [20,23]. Furthermore, *NPM1*^mut^ creates a specific epigenetic dependency on the KMT2A-Menin complex to maintain the overexpression of HOX and MEIS1 genes [24,25,26]. This unique epigenetic landscape not only drives leukemic self-renewal but also provides a specific vulnerability that can be targeted by emerging epigenetic therapies including revumenib and ziftomenib [27,28].

## 4. Epigenetics in AML

**Figure 1:**
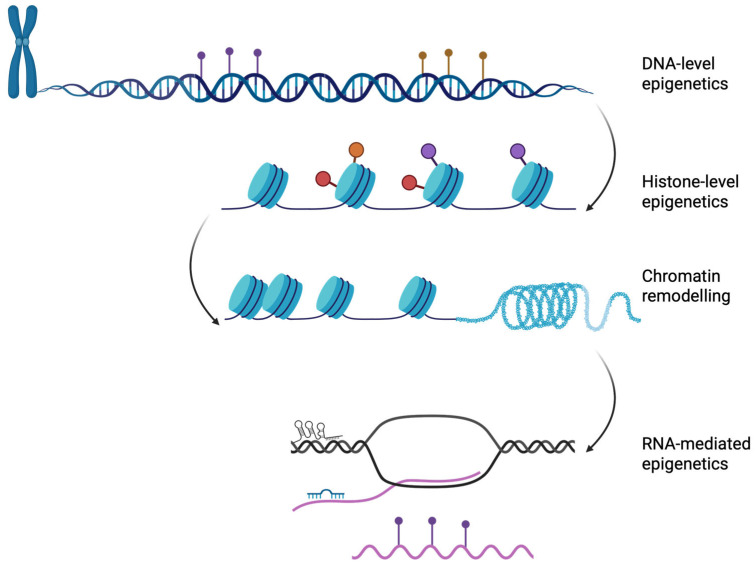
Schematic representation of different levels of epigenetic regulation. At the DNA level, epigenetic modifications such as DNA methylation and hydroxymethylation occur directly on the DNA strand, influencing gene expression. At the histone level, histone proteins undergo post-translational modifications such as acetylation and methylation, which affect chromatin structure and transcriptional activity. Chromatin remodeling involves nucleosome repositioning and changes in chromatin compaction, regulating the accessibility of DNA to transcription factors and other regulatory proteins. At the RNA level, RNA modifications such as methylation and non-coding RNAs influence transcription, RNA stability, splicing, and translation.

Epigenetics is the modification of gene expression without alterations to the DNA sequence itself, representing a fundamental mechanism that regulates cellular development and lineage identity [29]. Broadly, epigenetic mechanisms can be categorized into four interconnected groups including modifications occurring directly on the DNA molecule, post-translational changes to histone proteins, chromatin remodeling, and regulation by RNA molecules (Figure 1; Table 1). In AML, these epigenetic alterations play a pivotal role by disrupting the normal hematopoietic differentiation and self-renewal mechanisms, leading to an aberrant transcriptional program in AML [30].

DNA-level epigenetics primarily involves chemical modifications of DNA that regulate gene expression without altering the DNA sequence, mainly through processes of DNA methylation and DNA demethylation. Aberrant DNA methylation causes widespread transcriptional dysregulation and plays a central role in leukemogenesis [4,30]. Histone modifications, which regulate gene expression by post-translationally modifying histone proteins, represent another common type of epigenetic abnormality in AML. Histone modifications can influence chromatin structure and accessibility, often by recruiting or signaling to chromatin remodeling complexes. In turn, chromatin remodeling is further regulated by other mechanisms, such as ATP-dependent SWI/SNF complex, architectural proteins like CTCF and cohesin, and the incorporation of histone variants [35,36,37]. In addition, the epigenetic landscape is also modulated by RNA-based mechanisms, including non-coding RNAs and chemical modifications of RNA molecules. These processes play important roles in regulating gene expression and contribute to both normal hematopoiesis and malignant transformation. The RNA-based epigenetics also include post-transcriptional RNA modifications, which play crucial roles in regulating RNA stability, splicing, transport, and translation, thereby influencing gene expression and cellular function [38].

### 4.1. DNA-Level Epigenetics

In the eukaryotic genome, DNA methylation refers to the addition of a methyl group to the 5-carbon position of cytosine residues, resulting in the formation of 5-methylcytosine (5mC). 5mC predominantly occurs at CpG dinucleotides, whereas non-CpG methylation (CpA, CpT, CpC) has been reported, particularly in embryonic stem cells and neurons [41,42]. DNA methylation leads to gene repression or activation depending on the location of the methylation. When DNA methylation occurs in promoters or enhancers, it typically results in gene repression. For example, the hypermethylation of the *CDKN2A* promoter in AML results in the silencing of this critical cell cycle inhibitor and is associated with poor prognosis [43,44]. The function of DNA methylation within gene bodies remains incompletely understood. This methylation was initially considered a mechanism for silencing repetitive DNA elements. In contrast, several studies have reported that gene body methylation is associated with active transcription. The precise mechanism remains debated and appears to be highly context-dependent, possibly involving the regulation of alternative splicing or the suppression of cryptic promoter activity within gene bodies [42,45,46].

DNA methylation is catalyzed by a family of DNA methyltransferases, including DNMT1, DNMT3A, and DNMT3B, which act as writers to install methyl groups on cytosine residues [47]. DNMT3A and DNMT3B are responsible for establishing de novo methylation patterns on previously unmethylated DNA, and DNMT1 primarily maintains existing methylation patterns during DNA replication by copying the methylation marks from the parental DNA strand onto the daughter strand [48]. In addition, DNMT1 can also function as a de novo methyltransferase under certain conditions, such as in mouse oocytes lacking Stella and in the regulation of transposable elements in mouse embryonic stem cells [49,50].

Other human DNMTs include DNMT2 and DNMT3L. DNMT2, although structurally related to DNA methyltransferases, is not involved in DNA methylation, and instead functions as a tRNA methyltransferase. DNMT3L is closely related to this family but lacks intrinsic methyltransferase activity. Instead, it acts as a cofactor for DNMT3A and DNMT3B, enhancing their de novo DNA methylation activity [48]. Additionally, DNA methylation is also interpreted and regulated by reader proteins, such as the MBD family and UHRF proteins. These readers recognize and bind to methylated DNA and subsequently recruit chromatin-modulating complexes that influence gene expression regulation [51].

**Figure 2:**
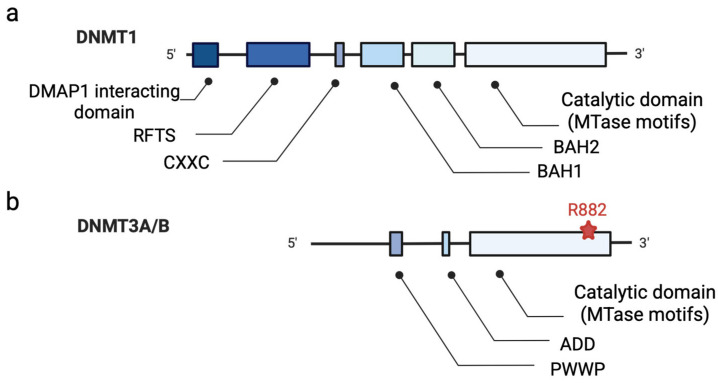
Structure of DNA methyltransferase including DNMT1 protein and DNMT3A/B protein. DNMT1 (**a**) and DNMT3A/B (**b**) share a conserved C-terminal catalytic methyltransferase (MTase) domain. DMAP1, DNA methyltransferase 1-associated protein interacting domain; RFTS, Replication foci targeting domain; CXXC, CXXC domain; BAH1 and BAH2—bromo-adjacent homology domains 1 and 2. PWWP, Pro-Trp-Trp-Pro domain; ADD, ATRX–DNMT3–DNMT3L domain. Red star indicates the most common types of mutation in DNMT3A [48].

Structurally, both DNMT1 and DNMT3A/B share a conserved catalytic methyltransferase domain at the C-terminus, which is responsible for mediating methyl group transfer. In their N-terminal regulatory domains, DNMT1 contains several unique features compared to DNMT3A/B, including a binding domain for DNA methyltransferase 1-associated protein 1 (DMAP1), a replication foci targeting domain (RFTD), and a CXXC domain that recognizes unmethylated CpG sites (Figure 2). In addition, it harbors two bromo-adjacent homology (BAH) domains, which emerging evidence suggests contribute to the recognition of histone H4K20me3 and play a role in mediating de novo methylation in vivo [48,52,53]. In contrast to DNMT1, DNMT3A and DNMT3B possess distinct N-terminal domains, including a protein–protein interaction domain (PWWP) and a histone H3 recognition domain (ADD) [48].

DNA methylation has been extensively studied in both normal hematopoiesis and leukemogenesis. In healthy hematopoietic cells, it is tightly regulated to ensure proper lineage commitment and differentiation. In AML, however, this regulation is disrupted, resulting in widespread epigenetic reprogramming. These alterations give rise to distinct DNA methylation signatures that can be used to define the biological subtypes of AML [54]. Aberrant DNA methylation in AML can arise from mutations in DNMTs or alterations in their expression levels. Within the DNMT family, DNMT3A mutations are the most common, occurring in about 22% of AML cases, while mutations in DNMT1 and DNMT3B are rare. The most common DNMT3A mutation occurs at the R882 residue within its catalytic domain (Figure 2), disrupting normal de novo methylation activity and leading to localized aberrant methylation patterns that are associated with poor prognosis and therapeutic resistance [23,55]. Beyond mutations, DNMT1, DNMT3A, and DNMT3B are often upregulated in AML, contributing to widespread epigenetic dysregulation [56]. This aberrant methylation landscape represents a targetable epigenetic vulnerability, underlying the sensitivity of epigenetically dysregulated AML to hypomethylating agents [57].

In parallel with DNA methylation, DNA can also undergo active demethylation, a process primarily initiated by the TET family of enzymes. These enzymes catalyze the oxidation of 5-methylcytosine (5mC) to 5-hydroxymethylcytosine (5hmC), which represents the first step of active DNA demethylation and is typically associated with transcriptional activation. Mutations in TET2 are frequently observed in AML, leading to disruption of this process and contributing to leukemogenesis [4,30]. In addition to the TET family of proteins, other enzymes can also contribute to DNA demethylation. For instance, AID/APOBEC enzymes can deaminate cytosine or methylated cytosine to uracil, generating a mismatch that is subsequently recognized and excised by thymine DNA glycosylase (TDG). The base excision repair (BER) machinery then replaces the altered base with an unmodified cytosine. This additional process is well-characterized in other biological systems, such as B-cells, but mutations in AID/APOBEC or TDG are rarely detected in AML [32,33]. Consequently, current studies of DNA demethylation in AML have primarily focused on the TET family of enzymes.

DNA demethylation is also influenced by metabolic enzymes including the IDH family. Mutations in IDH1 or IDH2 frequently occur in AML, accounting for approximately 20% of AML cases. Under normal conditions, IDH enzymes are responsible for catalyzing the production of α-ketoglutarate (α-KG), an essential cofactor for α-KG-dependent dioxygenases including TET enzymes. IDH mutations instead produce 2-hydroxyglutarate (2-HG), which acts as a competitive inhibitor of α-KG, thereby blocking TET enzyme activity and ultimately inhibiting DNA demethylation [58,59].

Clinically, these DNA-level epigenetic alterations in AML carry distinct prognostic and predictive weight, yet they differ markedly in their therapeutic actionability. For *DNMT3A*-mutated AML, no *DNMT3A*-mutated-directed therapy exists. The evidence-based standard remains intensive chemotherapy for fit patients and hypomethylating-agent-based regimens for those ineligible for intensive chemotherapy. Similarly, no *TET2*-directed agent is available, and these patients receive the same standard regimens [15]. In contrast, *IDH1* and *IDH2* are actionable epigenetic-pathway mutations with evidence-based targeted therapies, including ivosidenib (*IDH1*) and enasidenib (*IDH2*), with additional combination regimens under active investigation [60,61,62].

### 4.2. Histone-Level Epigenetics

Histone modification is another major form of epigenetic regulation in AML. These modifications mainly include acetylation, methylation, phosphorylation, ubiquitination, SUMOylation, deimination, and ADP-ribosylation, each defined by the specific chemical group added or altered on histone residues [34]. Among those, methylation is one of the most extensively studied and is particularly relevant in AML pathogenesis. Histone methylation is catalyzed by histone methyltransferases, which add a methyl group to specific lysine residues on histone tails. In contrast, these marks can be primarily removed by histone demethylases belonging to the lysine demethylase (KDM) family. Each lysine can be mono-, di-, or tri-methylated, with the degree of methylation providing an additional layer of regulation.

Histone methylation can function as either a transcriptional activation or repression signal, depending on the specific residue that is methylated and the extent of methylation [63,64]. Activation-associated methylation marks typically include H3K4me3, H3K36me3, and H3K79me3, whereas repressive marks primarily include H3K9me3, and H3K27me3. H4K20 methylation (me1/2/3) has been associated with both activation and repression depending on the methylation state and genomic context (Table 2). Misregulation of histone methylation is a key epigenetic mechanism driving AML pathogenesis, arising from mutations, chromosomal translocations, or altered expression levels of those histone methyltransferases and demethylases. Among these, *KMT2A*-r AML represents one of the best-characterized examples, as described previously. Other examples include DOT1L-mediated dysregulation of H3K79 methylation, leading to overexpression of leukemogenic genes such as HOXA9, and upregulated expression of SETDB1, which drives aberrant accumulation of H3K9me3 and results in stable repression of tumor suppressor and differentiation-associated genes [65,66].

**Figure 3:**
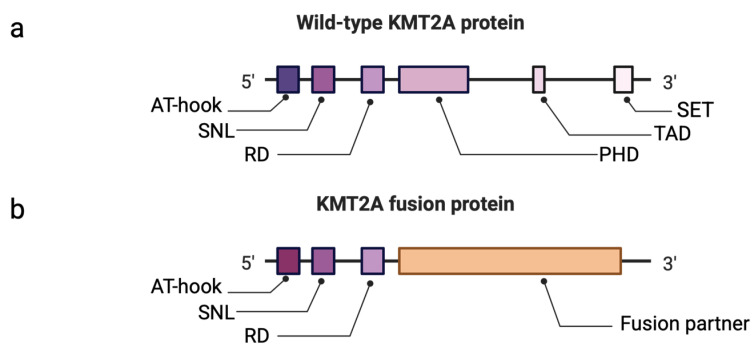
Structure of the WT and fusion forms of the KMT2A protein. Schematic representation showing the domain organization of the WT KMT2A protein (**a**) and its rearranged fusion form (**b**). The WT KMT2A protein contains several conserved domains, including the AT-hook motifs, the speckled nuclear localization (SNL) signal, the repression domain (RD), and a cluster of plant homeodomain (PHD) fingers, a transcriptional activation domain (TAD), and a H3K4 histone methyltransferase domain (SET). Chromosomal translocations involving *KMT2A* result in KMT2A fusion proteins in which the C-terminal domains, including PHD, TAD and SET, are replaced by various fusion partners [67,68].

A well-characterized example of such epigenetic dysregulation involves the KMT2A gene (also known as MLL1), which encodes a histone methyltransferase with multiple functional domains that mediate chromatin modification and transcriptional regulation. It specifically catalyzes the methylation of histone H3 at lysine 4 (H3K4) to promote transcriptional activation. KMT2A also interacts with RNA Polymerase II and other regulatory complexes to facilitate gene expression and maintain the epigenetic programs essential for cell identity and differentiation [67]. The N-terminal region of KMT2A contains multiple AT-hook motifs that facilitate binding to AT-rich DNA sequences, two speckled nuclear localization signals, and two repression domains (RDs) that recruit interacting partners. The protein also harbors a cluster of plant homeodomain (PHD) fingers involved in protein–protein interactions and chromatin recognition, a transcriptional activation domain (TAD), and a SET domain at the C-terminus that catalyzes H3K4 methylation (Figure 3a) [67,68]. WT KMT2A is essential for normal embryonic development and hematopoiesis. Genetic deletion of *Kmt2a* in murine models results in embryonic lethality, accompanied by severe defects in fetal hematopoiesis and impaired differentiation of hematopoietic stem and progenitor cells [69,70]. Chromosomal translocations, deletions, and inversions that result in gene rearrangements are among the best-characterized initiating events in leukemogenesis and represent major driver alterations in AML. *KMT2A* is a representative gene frequently involved in chromosomal rearrangements in AML, which accounts for approximately 3% of AML cases and is associated with adverse risk [15,22]. KMT2A fusion proteins lack several key functional domains present in the WT protein, most notably those in the C-terminal region, including the SET domain, the TAD and the PHD (Figure 3b).

Over 100 KMT2A translocation partners have been identified in *KMT2A*-rearranged (*MLL*-rearranged) leukemia. Across acute leukemias, the most frequent partners—AFF1 (AF4), MLLT3 (AF9), MLLT1 (ENL), MLLT10 (AF10), AFDN (AF6), and ELL—together with KMT2A partial tandem duplications (PTDs) account for the large majority of cases [71]. Among them, AF9 is the most prevalent fusion partner of *KMT2A* in AML, representing approximately 30% of *KMT2A*-rearranged AML cases. AF9 is a transcriptional cofactor that promotes gene expression and regulates chromatin by interacting with elongation complexes and multiple chromatin-modifying proteins, including the histone methyltransferase DOT1L. WT AF9 is essential for governing the self-renewal capacity of HSCs and progenitor cells [72,73]. The chromosomal rearrangement of *KMT2A* and *AF9* results in the combination of the N-terminal domain of KMT2A and the C-terminal domain of AF9. This generates a fusion protein that interacts with transcriptional activators and epigenetic regulators, aberrantly activating gene expression programs associated with leukemogenesis, particularly members of the *HOXA* cluster and their cofactor *MEIS1* [67]. Persistent overexpression of *HOX* genes and *MEIS1* is a hallmark of *KMT2A*-rearranged AML and plays a central role in sustaining leukemia cell self-renewal and proliferation [74]. In addition, maintenance of this *HOXA*/*MEIS1* program requires the menin–KMT2A interaction, rendering *KMT2A*-rearranged AML a paradigmatic example of a targetable epigenetic vulnerability that can be exploited by menin inhibition. The parallel dependence on DOT1L-mediated H3K79 methylation likewise represents a targetable axis, although DOT1L inhibitors have shown more limited clinical activity to date [27,75].

Acetylation is another common type of histone modification, in which an acetyl group is added to specific lysine residues on histone tails. This process is catalyzed by a group of histone acetyltransferases (HATs) including the p300/CBP, MYST family such as MOZ, and GNAT family such as GCN5. Conversely, histone acetyl groups can be removed by histone deacetylases (HDACs), including classical HDACs and sirtuins. Common histone acetylation sites include H3K9ac, H3K14ac, H3K18ac, H3K27ac, H3K56ac, H4K5ac, H4K8ac, and H4K12ac, which are generally associated with active gene transcription [76,77]. Dysregulation of histone acetylation occurs through aberrant activity of histone acetyltransferases (HATs) or histone deacetylases (HDACs). For example, overexpression of p300/CBP leads to aberrant epigenetic and transcriptional regulation that drives AML pathogenesis and represents a potential therapeutic target for epigenetic therapy [78].

While histone methylation and acetylation are the most extensively studied in AML, other modifications such as ubiquitination, SUMOylation, deimination and ADP-ribosylation also play important roles. For example, H2B ubiquitination plays a vital role in regulating DOT1L activity in AML by promoting nucleosome binding and H3K79 methylation necessary for leukemic gene expression [79]. In another case, PARP-mediated ADP-ribosylation contributes to the sensitivity of AML cells to chemotherapy [80]. Similarly, the first-in-class SUMOylation inhibitor (TAK-981) demonstrates potent anti-leukemic activity in AML models [81].

Clinically, the menin–KMT2A axis is one of the most clinically actionable histone-methylation targets in AML, with *KMT2A* rearrangement and *NPM1* mutation serving as the defining biomarkers of likely benefit from menin inhibitors [27]. The principal barrier for menin inhibitors is durability rather than initial response, as acquired on-target *MEN1* mutations and other adaptive mechanisms drive relapse [82]. Although *KMT2A*-rearranged AML also depends on DOT1L-mediated H3K79 methylation, the DOT1L inhibitor pinometostat showed only modest single-agent clinical activity and remains investigational [75]. Additional histone-modifying enzymes are also being targeted clinically such as LSD1 histone demethylase inhibitors which have entered phase 1/2 trials in AML [83].

### 4.3. Chromatin Remodeling

Histone modification, such as methylation and acetylation, can influence chromatin accessibility, but chromatin structure is also regulated by other mechanisms that directly remodel the nucleosome landscape and organize higher-order chromatin architecture. These processes are critical for maintaining genome stability and orchestrating precise epigenetic and genetic programs. A representative example of this is ATP-dependent chromatin remodeling complexes including the SWI/SNF family and INO80. Previous studies have shown that loss-of-function mutations in SWI/SNF components, such as ARID1A, ARID2, and SMARCA2/4, frequently occur in AML, leading to impaired nucleosome remodeling and blockage of normal hematopoietic differentiation [84]. Other chromatin remodeling mechanisms are also implicated in AML pathogenesis. For example, mutations in cohesin complex genes are present in a significant proportion of AML cases, with STAG2 mutations occurring in approximately 5–10% of patients. STAG2 mutations lead to disrupted chromatin looping and loss of proper domain boundaries, which causes misregulation of gene expression and ultimately drives leukemic transformation [85].

### 4.4. RNA-Mediated Epigenetics

The epigenetic landscape is also modulated by RNA-based mechanisms, including non-coding RNAs and chemical modifications of RNA molecules. These processes play important roles in regulating gene expression and contribute to both normal hematopoiesis and malignant transformation. Non-coding RNAs mainly include microRNAs, PIWI-interacting RNAs (piRNAs), small interfering RNAs (siRNAs) and long non-coding RNAs (lncRNAs). These molecules regulate gene expression through transcriptional control, modulation of mRNA stability, and chromatin remodeling [38]. The RNA-based epigenetics also include post-transcriptional RNA modifications, such as the addition of a methyl group to the nitrogen-6 position of adenosine within RNA molecules (m^6^A). This modification is primarily regulated by three groups of proteins: writers, such as METTL family proteins, which install the methyl group; readers, including YTH domain-containing proteins, which recognize and interpret the modification; and erasers, such as FTO and ALKBH5, which remove it. These mechanisms are essential for regulating RNA stability, transport, splicing, and translation, and their dysregulation has been linked to abnormal hematopoiesis and leukemogenesis [38]. Clinically, RNA modification is the most recently implicated epigenetic layer. One representative example is the METTL3 inhibitor STC-15, which has reached phase 1 clinical testing in solid tumors, with AML as an intended future indication supported by preclinical data [86].

### 4.5. Therapeutic Options for AML

AML treatment remains challenging because of the heterogeneous nature of the disease, characterized by diverse genetic and epigenetic abnormalities, which contributes to frequent relapse and poor long-term survival outcomes. Over the past several decades, the management of AML has relied heavily on intensive chemotherapy and allogeneic hematopoietic stem cell transplantation (HSCT) as the cornerstone of therapy. Patients diagnosed with AML first undergo an induction phase, which typically involves intensive chemotherapy designed to rapidly reduce leukemic blasts and achieve complete remission (CR). The standard chemotherapy agents used in this phase include cytarabine and anthracycline (such as daunorubicin or idarubicin). Cytarabine inhibits DNA synthesis, while anthracyclines induce DNA damage, and both ultimately trigger programmed cell death in rapidly dividing leukemic cells. The most widely used induction protocol is the 7 + 3 regimen (commonly referenced in the USA), which consists of 7 days of continuous infusion of cytarabine combined with 3 days of intravenous anthracycline. Once remission is achieved, treatment proceeds to the consolidation phase to prevent relapse, typically through high-dose or intermediate-dose cytarabine, depending on patient fitness, and/or allogeneic HSCT for those with higher-risk disease [15].

#### 4.5.1. FDA-Approved Targeted Therapies

While standard chemotherapy and HSCT have been used as fundamental therapies for AML patients, these treatment options remain significantly toxic, and their efficacy varies across different AML subtypes. Advances in genomic profiling have provided a deeper understanding of the molecular landscape of AML, which has facilitated the development of targeted therapies aimed at specific molecular drivers of the disease, offering the potential for more effective and less toxic treatment strategies. As of May 2026, FDA-approved targeted agents for AML fall into six mechanistic categories—FLT3 inhibitors, IDH inhibitors, BCL-2 inhibitors, Hedgehog pathway inhibitors, menin inhibitors, and CD33-directed antibody–drug conjugates—with approved indications and pivotal trials summarized in Table 3. These agents differ in their approved use: gilteritinib, ivosidenib, enasidenib, revumenib, and ziftomenib are approved as monotherapy in defined molecular subsets, whereas midostaurin, quizartinib, venetoclax, and glasdegib are approved only in combination with chemotherapy or hypomethylating agents. Gemtuzumab ozogamicin is approved for CD33-positive AML both as monotherapy and in combination with chemotherapy [87,88].

FLT3 mutations occur in approximately 30% of AML cases, with the most common type being FLT3-ITD (internal tandem duplication) and the less common type FLT3-TKD (tyrosine kinase domain mutation). This high frequency makes FLT3 a critical therapeutic target in AML. FLT3 inhibitors such as midostaurin, gilteritinib, and quizartinib are small-molecule tyrosine kinase inhibitors that bind to the mutant FLT3 receptor, blocking its kinase activity and thereby inhibiting downstream signaling pathways that drive leukemic cell proliferation and survival [89]. IDH mutations are another relatively frequent class of genetic alterations in AML. IDH inhibitors, such as ivosidenib (IDH1 inhibitor) and enasidenib (IDH2 inhibitor), directly target mutant IDH enzymes, leading to a reduction in 2-HG levels and the restoration of normal differentiation in leukemic cells. These agents have demonstrated clinical efficacy and are approved by the FDA for the treatment of patients with IDH-mutated AML [58].

In addition to agents targeting specific mutations, several therapies in AML focus on proteins that are aberrantly overexpressed or pathways that are hyperactivated. For example, BCL-2, an anti-apoptotic protein, is frequently overexpressed in AML, where it plays a key role in blocking the apoptotic pathway. Venetoclax, a selective BCL-2 inhibitor, competitively binds to BCL-2, displacing pro-apoptotic proteins such as BAX and BAK. This displacement restores the downstream apoptotic cascade, ultimately leading to programmed cell death in AML cells [90]. Similarly, overactivation of Hedgehog ligands or receptors has been identified as another therapeutic target in AML, where aberrant Hedgehog signaling plays a critical role in supporting the survival of leukemic stem cells (LSCs). Glasdegib, a Smoothened (SMO) receptor inhibitor, blocks Hedgehog pathway signaling by targeting the SMO receptor. When combined with low doses of cytarabine, glasdegib has been shown to inhibit LSC survival and is approved for use in older or unfit AML patients who are not eligible for intensive chemotherapy [91].

Moreover, targeted therapies can also act by disrupting the interaction partners of key molecular drivers of AML. Menin is a representative example of such a target. It is a scaffold protein that interacts with KMT2A fusion proteins and mutant NPM1. Disruption of this interaction by Menin inhibitors can suppress the downstream expression of leukemogenic transcription factors, thereby inhibiting AML cell survival [92]. Finally, antibody–drug conjugates (ADCs) represent another targeted strategy that combines monoclonal antibodies with cytotoxic agents. This approach increases the specificity of chemotherapy delivery by directing the cytotoxic drug specifically to AML cells, thereby enhancing efficacy and reducing systemic toxicity. A representative example is gemtuzumab ozogamicin, which consists of a monoclonal antibody targeting CD33, a well-established surface marker expressed on the majority of AML blasts [93].

#### 4.5.2. Epigenetic Therapies

As AML is characterized not only by genetic mutations but also by widespread epigenetic dysregulation, targeting epigenetic mechanisms has emerged as a promising therapeutic approach. Several epigenetic agents used in AML have been approved by the FDA or are currently in clinical trials. These therapies can be broadly classified according to the specific epigenetic mechanisms they target (Table 4). Among these, DNMT inhibitors including azacitidine and decitabine, were the first epigenetic therapies approved for clinical use in hematologic malignancies. They target DNMT1, DNMT3A, and DNMT3B, leading to DNA hypomethylation. These drugs have been primarily used in older or unfit patients who are unable to tolerate intensive chemotherapy, and are often combined with molecularly targeted agents. For instance, combining DNMT inhibitors with the BCL-2 inhibitor venetoclax has redefined upfront therapy for older individuals by simultaneously inducing hypomethylation and restoring downstream apoptotic cascades [94]. Despite high initial response rates, resistance to HMA plus venetoclax frequently develops and limits long-term efficacy, driven by the upregulation of alternative anti-apoptotic pathways, metabolic adaptation, or the acquisition of distinct subclonal mutational profiles [95]. IDH1/2 inhibitors are another promising class of therapies that indirectly affect DNA methylation and have shown encouraging outcomes, particularly when used in combination with DNMT inhibitors in patients with IDH-mutant AML [60].

In addition to targeting DNA-level epigenetic mechanisms, therapies aimed at histone-modifying enzymes are also being actively explored in AML. These agents can be broadly categorized based on their molecular function, including histone acetyltransferase inhibitors, histone deacetylase inhibitors, histone methyltransferase inhibitors, histone demethylase inhibitors, and histone acetylation reader inhibitors (Table 4). Beyond targeting histone-modifying enzymes directly, other epigenetic agents act on the chromatin-regulatory machinery, including drugs targeting menin and IDH1/2. Menin inhibitors disrupt this interaction and block the downstream oncogenic signaling in both *NPM1*-mutated and *KMT2A*-rearranged AML [27,28]. IDH1/2 inhibitors act on a mutant metabolic enzyme but produce an epigenetic effect, lowering oncometabolite 2-hydroxyglutarate to restore TET-mediated DNA and histone demethylation [96,97]. Additionally, multiple histone-modifying enzymes and chromatin readers are being targeted clinically, including HDAC inhibitors (e.g., panobinostat), BET bromodomain inhibitors (e.g., GSK525762), p300/CBP inhibitors (e.g., CCS1477), and PRMT5 inhibitors (e.g., GSK3326595), most of which have entered early-phase trials in AML, though several programs have since been discontinued and none has been approved for AML (Table 4). RNA modification inhibitors, particularly those targeting METTL3, have recently entered early-phase clinical development, with AML a leading intended indication, representing a novel therapeutic direction [86]. Overall, while DNMT and IDH1/2 inhibitors remain the only FDA-approved epigenetic therapies for AML, other classes of epigenetic agents represent an active and highly promising area of research.

**Table 4:** Overview of different classes of epigenetic agents evaluated in clinical trials. The table lists major classes of epigenetic targets, their primary molecular targets, and representative agents that have reached clinical investigation. Most listed agents have been evaluated in AML trials; the exceptions are the EZH1/2 inhibitor valemetostat, which is approved in other hematologic malignancies but remains investigational in AML, and the METTL3 inhibitor STC-15, which is in early-phase trials in solid tumors with AML as an intended indication [60,75,86,94,97,98,99,100,101,102,103].

Class	Primary Targets	Representative Agents	Status in AML
**DNA methyltransferase (DNMT) inhibitors**	DNMT1, DNMT3A, DNMT3B	Azacitidine, Decitabine	Approved (backbone therapy)
**Isocitrate dehydrogenase inhibitor**	IDH1/2	Ivosidenib (IDH1), Enasidenib (IDH2)	Approved (*IDH1*- or *IDH2*-mutated AML)
**Histone acetyltransferase (HAT) inhibitors**	p300/CBP	CCS1477	Phase 1/2
**Histone** **deacetylase** **(HDAC) inhibitors**	HDACs	Panobinostat	Phase 1/2
**Histone methyltransferase (KMT/PRMTs) inhibitors**	PRMT5	GSK3326595	Phase 1/2 (discontinued)
	DOT1L	Pinometostat	Phase 1 (discontinued)
	EZH1/2	Valemetostat	Approved in other hematologic malignancies; investigational in AML
**Histone demethylase (KDM) inhibitors**	LSD1	Iadademstat	Phase 1/2
**Histone acetylation reader inhibitors**	BETs	GSK525762	Phase 1/2 (discontinued)
**Menin Inhibitors**	Menin	Revumenib, Ziftomenib	Approved (*KMT2A*-rearranged and *NPM1*-mutated AML)
**RNA modification inhibitors**	METTL3	STC-15	Phase 1 in solid tumors; AML intended, preclinically supported

#### 4.5.3. Immunotherapies

Immunotherapy in AML has emerged as another critical area of treatment, aiming to harness the immune system to eliminate AML cells. Current immunotherapy approaches under clinical investigation for AML include monoclonal antibodies, bispecific T-cell engagers (BiTEs), immune checkpoint inhibitors, CAR-T cell therapies, NK cell-based therapies, and cancer vaccines. The most well-established target for monoclonal antibodies is CD33, which is commonly exploited in antibody–drug conjugates as discussed earlier. BiTEs are an approach that redirect endogenous T cells to recognize AML cells using engineered dual-binding antibodies, particularly for patients with relapsed disease after HSCT [104]. Immune checkpoint inhibitors, including anti-PD-1, anti-PD-L1, and anti-CTLA-4 antibodies, work by blocking inhibitory immune pathways to enhance T-cell activity against AML blasts. Their effect has been limited in AML, largely due to the low neoantigen burden of AML cells and the highly immunosuppressive bone marrow microenvironment. Current clinical trials are therefore exploring combination strategies with chemotherapy or DNMT inhibitors, which have shown more promising results [105].

CAR-T therapies utilize patient-derived T cells that are genetically engineered to recognize specific surface antigens on AML blasts. The most common CAR-T targets in AML include CD33, CD123, and FLT3. Overall, this treatment remains in the early stages of clinical development and faces significant challenges, such as risks of severe toxicity [105]. NK cell therapy is another immunotherapeutic option for AML; it leverages the innate tumor-killing capacity of NK cells to eliminate AML cells. Early-phase clinical trials have shown promising efficacy, but challenges remain to be addressed, such as the limited persistence of NK cells in vivo [106]. Cancer vaccines represent a distinct immunotherapeutic strategy that primarily uses dendritic cells or leukemia-associated antigens to stimulate the patient’s immune system to target AML blasts. Several of these vaccines have been evaluated in clinical trials, but their clinical efficacy remains limited, and further optimization is required to improve their therapeutic potential [107].

In addition, epigenetic therapies can overcome immune evasion by increasing the expression of tumor antigen presentation genes and re-expressing leukemia-associated antigens, enhancing the immune response in AML treatment. For example, DNMT inhibition can derepress endogenous retroviral transcripts, which triggers a double-stranded-RNA–sensing interferon response (“viral mimicry”) and upregulates immune signaling [108], providing a mechanistic rationale for combining epigenetic agents with immunotherapy. Several such combinations have been or are being evaluated in clinical trials. For example, azacitidine with a PD-1 inhibitor improves median overall survival compared to historical monotherapy controls in relapsed/refractory AML [109]. Moreover, triplet regimens that add an immune-directed agent to a hypomethylating agent–venetoclax backbone have also entered clinical trials, with mixed results to date [110,111].

## 5. Conclusions

In summary, epigenetic dysregulation plays a key role in AML, acting at multiple levels from DNA to RNA, including DNA methylation, histone post-translational modifications, chromatin remodeling, and RNA-mediated regulation. AML cells exploit mutations and alter expression of epigenetic regulators to reshape their transcriptional landscape, sustaining leukemic self-renewal, blocking myeloid differentiation, and promoting resistance to therapy. So far, several promising epigenetic agents have been approved or are under clinical investigation, such as DNA methyltransferase inhibitors, IDH1/2 inhibitors, and menin inhibitors. Despite significant advances, treatment resistance and disease relapse remain major challenges in AML. A deeper understanding of epigenetic alterations and their interactions with genetic lesions will be essential for the development of more effective and durable therapies. Overall, targeting epigenetic vulnerabilities represents a promising avenue for improving clinical outcomes in AML.

## Figures and Tables

**Table 1:** Categories of epigenetic regulation. This table summarizes the four principal categories of epigenetic regulation, which include DNA-level epigenetics, histone-level epigenetics, chromatin remodeling, and RNA-mediated epigenetics, along with their subtypes and representative examples [4,30,31,32,33,34,35,36,37,38,39,40].

Category	Sub-Type	Examples/Mediators
**1. DNA-level epigenetics**	DNA methylation	DNMT1, DNMT3A, DNMT3B
	DNA demethylation	TET family proteins (TET1, TET2, TET3), AID/APOBEC, TDG
**2. Histone-level epigenetics**	Acetylation	Histone acetyltransferases (e.g., p300/CBP) Histone deacetylases (e.g., HDAC1, HDAC2, HDAC3)
	Methylation	Histone methyltransferases (e.g., EZH2, KMT2A), Histone demethylases (e.g., LSD1, KDM)
	Phosphorylation	MSK1, MSK2
	Ubiquitination	RNF20/40
	SUMOylation	PIASs
	ADP-Ribosylation	PARPs
	Deimination	PADI4
**3. Chromatin remodeling**	ATP-dependent Chromatin remodellers	SWI/SNF complex, INO80
	Architectural proteins	Cohesin complex
	3D organization	TADs
	Histone variants	H2A.Z, H3.3
**4. RNA-mediated epigenetics**	Non-coding RNAs	microRNAs, PIWI-interacting-RNAs, siRNAs, long non-coding RNAs (e.g., MALAT1)
	RNA modifications	Writers (e.g., METTL3/14), Readers (e.g., YTH domain proteins), Erasers (e.g., FTO, ALKBH5)

**Table 2:** Key histone methylation sites, their associated enzymes, and primary functions. This table summarizes major histone methylation modifications relevant to gene regulation, along with their corresponding methyltransferases and demethylases [63,64].

Modification Site	Methyltransferases Example	Demethylases Example	Primary Functions
**H3K4me1/2/3**	MLL1-4 (KMT2A–D), SET1	KDM5, LSD1/2 (KDM1A/B)	Associated with transcriptional activation
**H3K9me1/2/3**	SETDB1, Suv39H1/2, G9a	KDM3/4/7	Associated with transcriptional repression
**H3K27me1/2/3**	EZH1/2	KDM6A-C	Associated with transcriptional repression
**H3K36me1/2/3**	SETD2/3, NSD1/2/3	KDM2A/B, KDM4A-D	Associated with transcriptional activation
**H3K79me1/2/3**	DOT1L	No confirmed eraser	Associated with transcriptional activation
**H4K20me1/2/3**	SET8, SUV4-20H1/2	KDM7B, PHF8	Associated with transcriptional activation/repression

**Table 3:** FDA-approved targeted therapies for AML. This table summarizes the six major classes of targeted agents currently approved by the FDA for the treatment of AML. Each class is categorized according to its primary molecular target, with representative drugs listed for each category [87,88].

Class	Primary Targets	Representative Agents
**FLT3 inhibitors**	FLT3 mutations (ITD and/or TKD)	Midostaurin, Gilteritinib, Quizartinib
**IDH inhibitors**	IDH1/2 mutations	Ivosidenib (IDH1), Enasidenib (IDH2)
**BCL-2 inhibitors**	BCL-2	Venetoclax
**Hedgehog Pathway Inhibitors**	Smoothened (SMO) receptor	Glasdegib
**Menin Inhibitors**	Menin	Revumenib, Ziftomenib
**Antibody–Drug Conjugates**	CD33	Gemtuzumab ozogamicin

## Data Availability

No new data were created or analyzed in this study. Data sharing is not applicable to this article.
